# Cas12a mediates efficient and precise endogenous gene tagging via MITI: microhomology-dependent targeted integrations

**DOI:** 10.1007/s00018-019-03396-8

**Published:** 2019-12-17

**Authors:** Pan Li, Lijun Zhang, Zhifang Li, Chunlong Xu, Xuguang Du, Sen Wu

**Affiliations:** 1 Beijing Advanced Innovation Center for Food Nutrition and Human Health, China Agricultural University, No. 2 Yuanmingyuan West Road, Beijing, 100193 China; 2 State Key Laboratory of Agrobiotechnology, College of Biological Sciences, China Agricultural University, No. 2 Yuanmingyuan West Road, Beijing, 100193 China; 3 Institute of Neuroscience, State Key Laboratory of Neuroscience, Key Laboratory of Primate Neurobiology, CAS Center for Excellence in Brain Science and Intelligence Technology, Shanghai Institutes for Biological Sciences, Chinese Academy of Sciences, Shanghai, 200031 China

**Keywords:** CRISPR, Genome editing, Homology-independent targeted integration, Negative selection, Sticky ends, Microhomology-dependent targeted integration

## Abstract

Efficient exogenous DNA integration can be mediated by Cas9 through the non-homology end-joining pathway. However, such integrations are often imprecise and contain a variety of mutations at the junctions between the external DNA and the genomic loci. Here we describe a microhomology-dependent targeted integration method, designated MITI, for precise site-specific gene insertions. We found that the MITI strategy yielded higher knock-in accuracy than Cas9 HITI for the insertion of external DNA and tagging endogenous genes. Furthermore, in combination with negative selection and four different CrRNAs targeting donor vectors and genome-targeted sites with a CrRNA array, MITI facilitated precise ligation at all junctions. Therefore, our Cas12a-based MITI method increases the repertoire of precision genome engineering approaches and provides a useful tool for various gene editing applications.

**Electronic supplementary material:**

The online version of this article (10.1007/s00018-019-03396-8) contains supplementary material, which is available to authorized users.

## Introduction

Site-specific transgene integration is primarily implemented by homology-directed repair (HDR) and non-homology end joining (NHEJ) pathways [1]. The precise introduction of foreign DNA to endogenous target sites through the HDR pathway is time-consuming and cumbersome as it requires the cloning of the homology arms for every gene. Also, since this method can only exert its effect in the S/G2 cell phase, its efficiency is low, especially in non-dividing cells [2]. Accurate site-specific gene insertions could also be mediated via a method called Obligate Ligation-Gated Recombination (ObLiGaRe) through the efficient NHEJ pathway using zinc-finger nucleases (ZFNs) or Tale nucleases (TALENs) [3], but its wide application is limited by both high cost and complicated design. CRISPR/Cas9 also enables convenient and efficient knock-in of external DNA cassettes at target sites in zebrafish [4, 5], mammalian cells [6–8], and so on via NHEJ pathway; however, the junctions between the external DNA and the genome usually contain various mutations. Several efforts have aimed to optimize this system, such as controlling the expression of Cas9 protein via fusion of FKBP12-L106P destabilization domain to Cas9 [9] and cleaving both donor and target sites with the same sgRNA via homology-independent target integration (HITI) strategy [10].

Cas12a, as a newly identified RNA-guided nuclease of the Cas12 family, has several distinct advantages in comparison with Cas9, such as lower mismatch tolerance and greater specificity [11, 12]. Cas12a can process its CRISPR RNAs (CrRNAs) array into mature CrRNAs, and this facilitates concurrent targeting of multiple genes, which distinguishes it from Cas9 [13, 14]. Another difference between these two nucleases is that Cas9 and Cas12a generate blunt and sticky ends, respectively [15, 16]. Cas12a mainly cuts at the 18th base site of the non-complementary strand and the 23rd base site of the complementary strand downstream of the PAM sequence, generating a 5-nt overhang when the spacer length is 20 nt or longer. When the spacer length is shorter than 20 nt, Cas12a mainly cleaves after the 14 nt on the non-complementary strand and the 22 nt on the complementary strand. This feature makes Cas12a a useful tool similar to restriction enzymes for precise in vitro DNA assembly [17, 18]. The blunt-ended DSB generated by Cas9 is compatible with the Gibson assembly method, which relies on sequence homology for in vitro molecular cloning [19, 20]. Based on these in vitro comparison studies, we hypothesized that Cas12a may be used in mammalian cells for precise gene knock-in, with better accuracy than Cas9.

In this study, we devised a Cas12a-based method for precise gene integration. In this method, the recognition site is readily engineered into the donor vector by a simple PCR or T4 ligation. The orientation of Cas12a target sequences in the donor vector must be opposite to that of the genome target, but the 5 base pairs distal to the PAM are identical to the last 5 bp sequences of the genome target site, which enables the generation of seamless junctions between endogenous and exogenous DNA via complementary sticky sequences. Since our new method depends on the 5 bp microhomology, we denoted it as microhomology-dependent targeted integration (MITI). MITI could increase the accuracy of knock-in outcomes compared with the Cas9 HITI approach at several loci. Therefore, MITI is a useful tool for precise targeting.

## Materials and methods

### Plasmid cloning

The related primer sequences of plasmid and CrRNA constructions, knock-in detection, and T7E1 assays used in this study are noted in Supplementary Tables S1–S4. To generate the SA-IRES-GFP donor vector, the pZGs plasmid [21] was digested with NcoI, removing the LacZ-CAG promoter and allowing self re-ligation. The SV40-Puro fragment was PCR amplified and inserted into the AflII/BglII-digested pZGs backbone through Gibson assembly. The CrRNA or sgRNA target sequence of Cas12a or Cas9, respectively, was inserted into the XhoI/SpeI*-*digested donor backbone before the SA sequence to construct the single-cut donor vector, including D1, D2, and D3. To construct the D7 vector containing the *HSV*-TK negative selection gene, the linker carrying the other *AAVS1* site CrRNA target sequence and the SalI enzyme cleavage site was inserted into the AscI/BglII-digested D2 backbone. A SalI/SpeI-digested TK1-Amp-ColE1 fragment from the pWS-TK7 plasmid was then ligated into the SalI/SpeI-digested vector backbone. Alternatively, donor vectors can be constructed by a simple PCR using the primers containing the target sequence to amplify the backbone fragment, followed by ligation with the reporter gene via Gibson assembly. The donor vector 3 × Flag-F2A-tdTomato-*lox*P-PGK-Puro-*lox*P was created through Gibson assembly, by combining the following five DNA fragments: (i) PCR-amplified 3 × Flag from pX330-U6-Chimeric_BB-CBh-hSpCas9 plasmid (Addgene 42230), (ii) PCR-amplified F2A fragment from pMaster12 plasmid (Addgene 58527), (iii) PCR-amplified tdTomato fragment from pRSET-B tdTomato plasmid, (iv) the PCR-amplified *lox*P-PGK-Puro-*lox*P fragment, and (v) NdeI/AscI-digested pPB-hNRAS^G12V^ [22] *piggyBac* backbone. The corresponding CrRNA target sequence was inserted into the NdeI/FseI-digested 3 × Flag-F2A-tdTomato-*lox*P-PGK-Puro-*lox*P vector to obtain the D4, D5, and D6 donor vectors. To construct the D4.1 vector, the linker containing the *CLTA* Cas12a target sequence identical to the genome was ligated into the AscI/AflII*-*digested D4 vector. The vector for expressing the AsCas12a CrRNA was constructed via linking the direct repeat sequence of AsCas12a and the BbsI enzyme site sequence into the BbsI/BamHI digested pCRISPR-sg4 backbone [22]. To get the all-in-one vector co-expressing the hU6-CrRNA and AsCas12a, the hU6-CrRNA (BbsI) PCR fragment was ligated into the NruI digested pY010 plasmid (Addgene 69982) backbone through Gibson assembly. The CrRNA array was constructed as previously described [14].

### Cell culture and transfection

HEK293T cells, HeLa cells, HepG2 cells and the primary pig fetal fibroblasts (PFFs) cells were all grown at 37 °C, in 5% CO_2_ in Dulbecco’s modified Eagle medium (DMEM) containing l-glutamine and pyridoxine hydrochloride supplemented with 10% heat-inactivated fetal bovine serum (FBS), 1 mM sodium pyruvate, 1 × NEAA and 1% penicillin and streptomycin. Cells were dissociated using 0.05% trypsin into single cells after washing in phosphate buffer saline and transfected using 4D-Nucleofector. For each transfection, 10^6^ cells were mixed with 100 μL pre-warmed nucleofection reagents. The cell suspensions were then mixed with plasmids and electroporated with a 4D-Nucleofector Device (Lonza). Transfected cells underwent puromycin selection for 5 days to ensure successful transfection and were then analyzed by flow cytometry (BD FACS Calibur).

### T7E1 assay

To prepare the genomic DNA from the cells for the T7E1 assay, cells were treated with 500 μL ES cells lysis buffer (100 mM NaCl; 20 mM Tris, pH 7.6; 10 mM EDTA; 0.5% SDS) and 15 μL of proteinase K (from 10 mg/mL stock) at 37 °C for 2–4 h without shaking. The genomic DNA was extracted as previously described [23]. Prior to PCR, 5 μL of genomic DNA was diluted in 15 μL HotShot lysis solution (25 mM NaOH, 0.2 mM EDTA), boiled for 15 min, and then neutralized with 15 μL of 40 mM Tris–Cl. The genomic region flanking the target site for each gene was PCR amplified using Q5 High-Fidelity DNA Polymerase (New England Biolabs, M0491S) following the manufacturer’s instructions. The 200–500 ng purified PCR fragments were mixed with 1 μL 10 × PfuUltra II Reaction Buffer (Agilent) and sterile ultra-pure water to a final volume of 10 μL and were subjected to a re-annealing process to enable heteroduplex formation: 95 °C for 10 min, 95 °C to 85 °C ramping at 2 °C/s, 85 °C to 25 °C at 0.25 °C/s, and 25 °C hold for 1 min. After re-annealing, the products were treated with 10 U of T7E1 enzyme (New England Biolabs) in 15 μL of the reaction mixture at 37 °C for 1 h, and the digested fragments were directly resolved on polyacrylamide gels. The Indel percentage was calculated with the following formula: 100 × (1 − sqrt (1 − (*b* + *c*)/(*a* + *b* + *c*))), where “*a*” represents the gray value of the intact band, and “*b*” and “*c*” are the gray values of each cleavage band. The gray values were determined using Image J software.

### Junction DNA sequencing by TA cloning

For junction sequence analysis after targeted knock-in, the genomic DNA of pooled cells or picked clones was extracted as described above. The target sites were PCR amplified using the Herculase II Fusion Enzyme (Agilent Technologies, Catalog #600677). For sequence analysis of the PCR product from pooled cells, GoTag polymerase (Agilent Technologies) was used to tail the PCR products, which were then cloned into the pMD19-T vector backbone (TakaRa, Catalog #6013) and sent for sequencing. For direct sequence analysis of the 5′ and 3′ junctions of picked clones, the PCR products were directly purified and sent for sequencing.

### Immunofluorescence staining

The HepG2 cells with stably integrated 3 × FLAG-2A-tdTomato reporter were washed with PBS and fixed in 4% paraformaldehyde for 30 min at room temperature. After washing three times in PBS every 5 min, the cells were blocked with blocking buffer (Beyotime Biotechnology, P0102) for 1 h at room temperature. The cells were then incubated overnight at 4 °C with the anti-FLAG-tag primary antibody (Beyotime Biotechnology, AF0036, 1:100). Next, the cells were incubated with the goat anti-rabbit IgG secondary antibody (Invitrogen, 1:1000) for 30 min in the dark after removing the primary antibody. Finally, DAPI (1:10,000) in PBS was added into the cells for 1 min. The immunostained cells were examined by fluorescence microscopy.

## Results

### Cas12a MITI strategy could facilitate more precise targeted integration

**Fig. 1:**
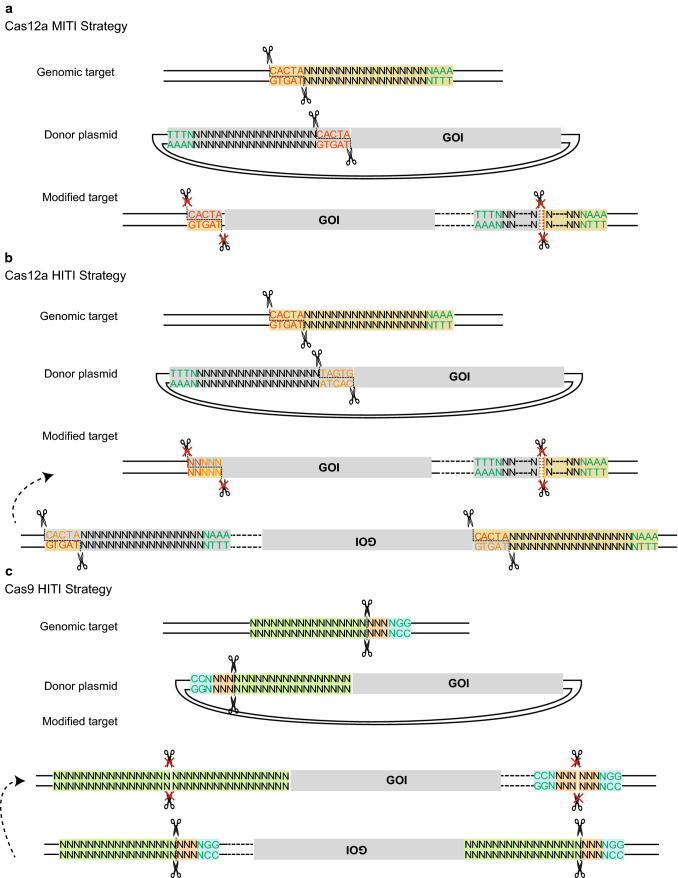
Schematic of Cas12a MITI, Cas12a HITI, and Cas9 HITI gene knock-in strategies. **a** Schematic of the Cas12a MITI system. The Cas12a MITI-based method needs to introduce modified compatible CrRNA target sequences in the donor. **b** Schematic of the Cas12a HITI system. The Cas12a HITI-based method requires a CrRNA target site in the donor in reverse orientation to the genomic target sequence resembling the Cas9 HITI approach. **c** Schematic of the Cas9 HITI system. The Cas9 HITI strategy requires an identical sgRNA target site in the donor but in reverse orientation to the genome target sequences. Green “TTTN” is the PAM sequence for the Cas12a. The gray scissors represents the Cas12a nuclease, and the black scissors represents the Cas9 nuclease

**Fig. 2:**
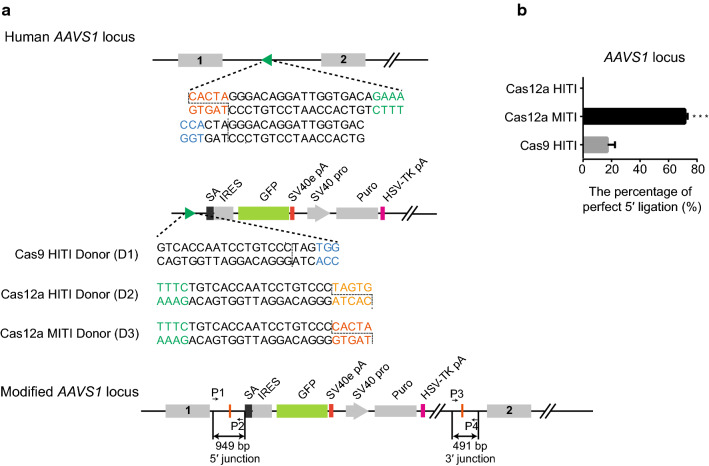
Comparison between the Cas12a HITI, Cas12a MITI, and Cas9 HITI strategies-mediated reporter knock-in at the *AAVS1* locus. **a** Schematic of the Cas9 HITI, Cas12a HITI and Cas12a MITI strategies at the *AAVS1* locus. The dotted lines represent the cleavage site of Cas9 or Cas12a. The sequence of the cleavage site is shown in vermilion for Cas12a MITI and in orange for Cas12a HITI. The protospacer adjacent motif (PAM) sequence of Cas12a is shown in green, and the PAM sequences of Cas9 are in blue. **b** The precise integration efficiencies of the three knock-in strategies at the *AAVS1* locus. The result was presented as mean ± SD, *n* = 3, *P < 0.05, **P < 0.01, ***P < 0.001, unpaired Student’s *t* test

To test the effect of Cas12a sticky overhangs on the outcomes of targeted integrations, we designed two simple strategies: Cas12a HITI and Cas12a MITI, which are different in that the 5 bp PAM-distal sequences are oriented in opposite directions (Fig. 1a, b). The sticky end generated by Cas12a on the donor plasmid in MITI is complementary with that of the endogenous target site. For comparison, the accuracy of the Cas9 HITI strategy was also examined (Fig. 1c). We next tested these strategies for targeting the reporter gene cassette—SA-IRES-GFP-SV40-Puro—into an editable *AAVS1* locus (Figs. 2a, S1). We first selected an overlapping target site at the *AAVS1* locus that can be cleaved simultaneously by Cas9 and Cas12a. We transfected HeLa cells with the corresponding Cas12a or Cas9 targeting vectors and donor plasmids and performed flow cytometry analysis for GFP-positive cells after 5 days of puromycin selection. After Sanger sequencing the TA-cloned 5′- and 3′-junction PCR products of the Cas12a HITI, Cas12a MITI, and Cas9 HITI HeLa cells, we found that 70% of Cas12a MITI-mediated integrations were accurate at the 5′ junctions, whereas precision of Cas9 HITI integrations was only 16.67%, and Cas12a HITI had no precise integrations (Figs. 2b, S2A-D). At the 3′ junction, precision of Cas12a MITI-mediated integrations was less accurate (Fig. S2E), possibly due to re-digestion by Cas12a after integration. In brief, these results suggested Cas12a MITI facilitates efficient precise targeted integration. It should be noted that potential large deletions of GFP were not considered when analyzing the above results. However, this should not bias our conclusions since all three methods were analyzed the same way.

### MITI allows for more efficient seamless reporter gene tagging

**Fig. 3:**
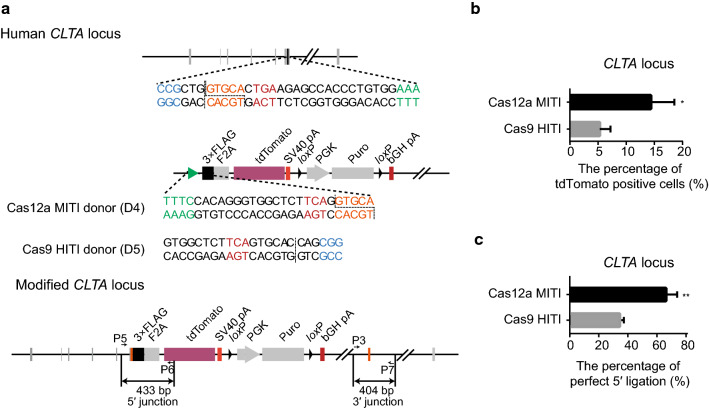
Tagging the *CLTA* gene using the Cas12a MITI or Cas9 HITI strategy in HEK293T cells. **a** Schematic of *CLTA* gene targeting using the two approaches. Red “TGA” base pairs are the termination codon of the *CLTA* gene. **b** Relative precise knock-in efficiency of the two strategies in HEK293T cells at the *CLTA* locus tested via the percentage of tdTomato positive cells evaluated by flow cytometry analysis. **c** The precise integration efficiencies of the two knock-in strategies at the *CLTA* locus via TA cloning and Sanger sequencing. All the results were presented as mean ± SD, *n* = 3, *P < 0.05, **P < 0.01, ***P < 0.001, unpaired Student’s *t* test

To compare the accuracy of Cas12a MITI and Cas9 HITI, we next used a 2A-tdTomato reporter system to tag a constitutively expressed gene *CLTA* in 293T cells (Fig. 3a). By flow cytometry analysis, we found that Cas12a MITI had a significantly higher percentage of tdTomato positive cells (14.40 ± 2.40%) than Cas9 HITI (5.30 ± 1.10%) (*P *= 0.026, Fig. 3b) after 5 days of puromycin selection. This low frequency of precise integration may be due to the existence of an identical target sequence for both Cas12a and Cas9 in the *CLTA* pseudogene on chromosome 12. The 5′-end integration junction of unsorted cells was amplified for sequence analysis, which showed that Cas12a MITI had a higher in-frame and precise integration rate than Cas9 HITI (Figs. 3c, S3). We further tested Cas12a MITI for targeting the silenced gene *GREB1L* with 2A-tdTomato in porcine fibroblasts (Fig. S4A). After transfection and puromycin selection, we picked 24 cell clones for PCR and sequencing analysis (Fig. S4B). We found that 91.67% of clones had the targeted integration at the 5′ junction and 81.82% of the integrations had the expected junction sequence (Fig. S4C). The above results further demonstrated that Cas12a MITI can be strategically used for more precise reporter gene targeting.

### Two cleavages in the donor help exclude unintended integration of the plasmid backbone

**Fig. 4:**
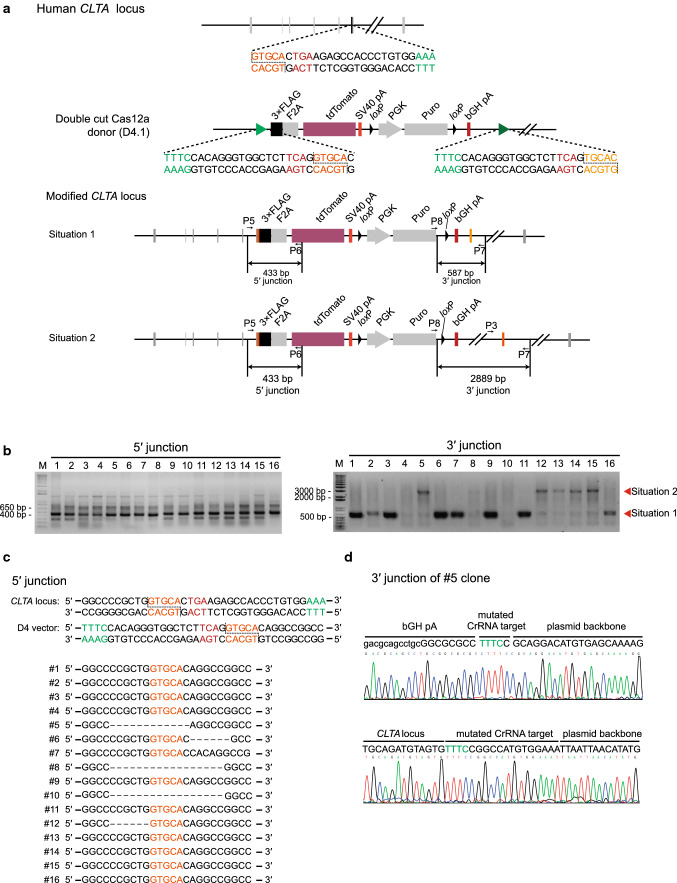
Tagging the *CLTA* gene using the two-cut donor in HepG2 cells. **a** Schematics of the donor plasmid and targeting strategy for CRISPR/Cas12a-mediated insertion of the 3 × FLAG-2A-tdTomato reporter at the *CLTA* locus in HepG2 cells. The two-cut donor (D4.1) includes two Cas12a recognition sites, one is the MITI-modified CrRNA target sequence, and the other is identical to the genomic target. The genetically modified *CLTA* locus may result in two different situations. One is that only the reporter part is knocked into the target site, and the other one is that the entire vector containing the prokaryotic backbone is integrated into the target site. **b** PCR identification of tdTomato-positive HepG2 clones. **c** Identification of tdTomato-positive HepG2 picked clones bearing predicted integration of 3 × FLAG-tdTomato by PCR and Sanger sequencing at the 5′ junction. **d** The sequences of the 3′ junction site of the representative HepG2 clone integrated with the whole D4.1 vector

To avoid integration of unwanted donor plasmid backbone, which may result in pronounced transgene silencing [10], a second Cas12a recognition sequence with perfect homology to the genome target sequence was added to the *CLTA* donor plasmid behind the puromycin gene (Fig. 4a). After transfection into HepG2 cells and 5 days of puromycin selection, we picked 16 tdTomato-positive clones. PCR and sequencing results showed 10 out of 16 tdTomato-positive cells had the predicted 5′ junction sequence (Fig. 4b, c), and 8 out of 16 clones were successfully targeted without the integration of the plasmid backbone (Fig. 4b, d). Further verification of these successful tagging events was confirmed with immunostaining (Fig. S5). These results indicate that Cas12a MITI can readily avoid plasmid backbone integration.

### Improved accuracy of both junctions with two cleavage sites on MITI donors

**Fig. 5:**
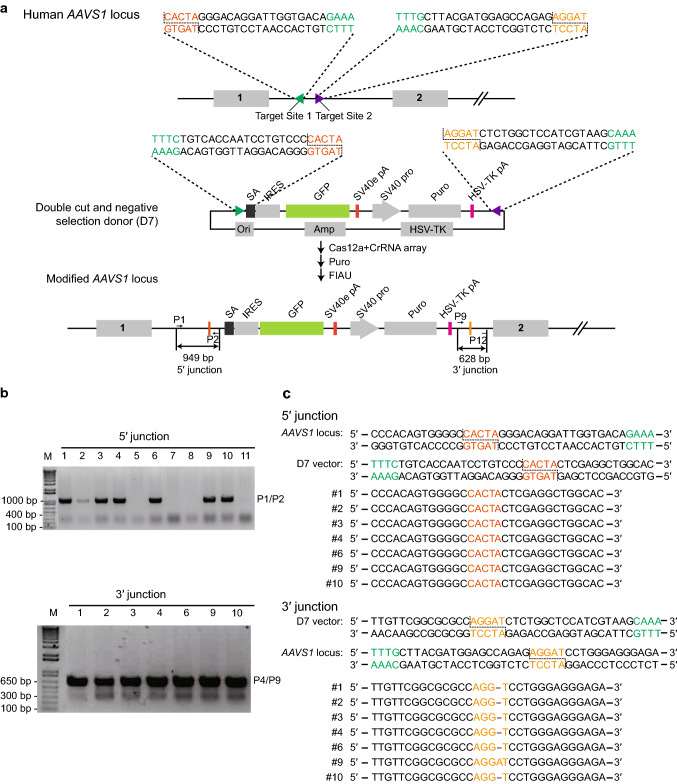
Two-cut MITI and negative selection strategy using Cas12a improved the accuracy of both junctions. **a** Schematics of the donor plasmid and targeting strategy for CRISPR/Cas12a-mediated insertion of the SA-IRES-GFP reporter at the *AAVS1* locus. The donor plasmid carries two modified Cas12a target sites at both the 5′ and 3′ sides of SA-IRES-GFP (two-MITI donor, D7) which correspond to the genomic target sites, and a negative selection gene herpes simplex virus thymidine kinase (*HSV*-*tk*) in the prokaryotic backbone of the donor. **b** Detection of targeted integration of SA-IRES-GFP cassette from puromycin and FIAU dual resistant clones via PCR. Seven out of eleven clones had expected integration at the left and right junctions. **c** The sequencing results of the 5′- and 3′-integration junctions amplified from GFP-positive clones produced by the two-MITI donor

To improve the sequence fidelity of both junctions between endogenous DNA and the exogenous gene, we selected two different neighboring genomic sites to target, and placed the corresponding MITI recognition sites around the foreign reporter gene in the donor vector. Meanwhile, we also introduced a herpes simplex virus thymidine kinase (*HSV*-*tk*) suicide cassette—a negative selection marker for avoiding random integration of the targeting vector [24, 25]—positioned outside of the reporter gene to exclude unintended genomic integration of donor plasmid (Fig. 5a). Two MITI cleavages on the donor plasmid eliminate the *HSV*-*tk* suicide gene along with cell sensitivity to Fialuridine (FIAU). After transfection and drug selection, we picked puromycin and FIAU dual resistant clones and analyzed both 5′ and 3′-end junction sequences by PCR sequencing. We found that in the puromycin and FIAU dual resistant clones, precise 3′ junction integration efficiency was improved (Fig. 5b, c). Our results suggest that two cleavage sites in combination with negative selection facilitate precise targeted integration of exogenous genes at both junctions. However, when using pairs of CrRNAs, complicated repair events may occur [26], and should be further examined in the future.

## Discussion

In this study, we demonstrated that sticky ends generated by Cas12a could facilitate the precise integration of foreign DNA fragments into the target genomic locus. The MITI method relies on Cas12a-induced stable complementary sticky overhangs which can enhance the efficiency and precision of in situ reporter gene targeting, and consequently greater fidelity of targeted gene expression. Our side-by-side comparison experiment revealed that the accuracy of end joining with MITI tends to be higher than the Cas9 HITI strategy at the different loci tested. MITI could be an alternative tool for future gene therapy applications. However, it should be noted that the method we used has some limitations. The number of colonies analyzed in the current study is still small. The results could be further strengthened using NGS.

Using a dual MITI strategy, we demonstrated the feasibility of precisely replacing a segment of DNA with the desired external DNA sequence via simultaneous cleavage of two neighboring sites in the genome and two corresponding target sites in the donor vector flanked by a negative selection cassette. In contrast, the Cas9 HITI strategy is currently capable of inserting foreign DNA into only a single genomic locus. This dual MITI strategy may be useful in certain situations, such as adding the *lox*P sequences to both sides of the exon for constructing conditional knockout animals or substituting the whole target gene with the desired coding sequences.

When tagging endogenous genes with a reporter, Cas9-mediated homology-independent knock-in via HITI always deletes the last few codons of the gene, destroying its integrity, which might have an impact on the function of the gene. For example, in our study, when the reporter cassette was used to tag the human *CLTA* gene using a Cas9 HITI approach, six base pairs were inevitably deleted (Fig. S3). Our MITI approach avoided such genetic deletion by adding essential base pairs after the cleavage site in the donor making it more flexible than the Cas9 HITI method.

Nonetheless, further examination of potential limitations and optimization of the MITI method is required. For example, widespread nickase activity of Cas12a [27–29] has been reported and may cause random insertion of foreign genes. However, since the fidelity of Cas12a is higher than Cas9 nuclease [11, 12], MITI is likely to have fewer off-target integrations than Cas9 HITI. Furthermore, when using pairs of CrRNAs for dual Cas12a targeting at two different loci, complicated repair events may occur, similar to that seen for CRISPR/Cas9, including the formation of extrachromosomal circular DNA, DNA inversion, and translocation [26]. This should be further tested and verified in the future. Considering there are many kinds of Cas12 with different specificities, potencies and PAM dependence [17, 30–33], it is possible to extend editable sequence space and enable further optimization of our MITI method.

### Electronic supplementary material

Below is the link to the electronic supplementary material.

Supplementary material 1 (PDF 9919 kb)

## Abbreviations

MITI: Microhomology-dependent targeted integration
HITI: Homology-independent targeted integration
CRISPR: Clustered regularly interspaced short palindromic repeats
CrRNAs: CRISPR RNAs
DSB: Double-strand break
NHEJ: Non-homology end joining
HDR: Homology-directed repair
GOI: Gene-of-interest
ZFNs: Zinc finger nucleases
ObLiGaRe: Obligate ligation-gated recombination
TALENs: Tale nucleases
PFFs: Pig fetal fibroblasts
*HSV*-*tk*: Herpes simplex virus thymidine kinase
