# Supplementary material for: Cas12a mediates efficient and precise endogenous gene tagging via MITI: microhomology-dependent targeted integrations
Source: Cell Mol Life Sci. 2019 Dec 17;77(19):3875–84. doi: 10.1007/s00018-019-03396-8 (PMC7508734; doi:10.1007/s00018-019-03396-8)
Supplement: Supplementary file 1 — Supplementary material 1 (PDF 9919 kb) [file 18_2019_3396_MOESM1_ESM.pdf]

**SUPPLEMENTAL MATERIAL**

# Cas12a mediates efficient and precise endogenous gene tagging via MITI: microhomology-dependent targeted integrations

Pan Li[1,2] · Lijun Zhang[1,2] · Zhifang Li[1,2] · Chunlong Xu[3] · Xuguang Du[1,2] · Sen Wu[1,2]

[1] Beijing Advanced Innovation Center for Food Nutrition and Human Health, China Agricultural University, No 2 Yuanmingyuan West Road, Beijing 100193, China

[2] State Key Laboratory of Agrobiotechnology, College of Biological Sciences, China Agricultural University, No 2 Yuanmingyuan West Road, Beijing 100193, China

[3] Institute of Neuroscience, State Key Laboratory of Neuroscience, Key Laboratory of Primate Neurobiology, CAS Center for Excellence in Brain Science and Intelligence Technology, Shanghai Institutes for Biological Sciences, Chinese Academy of Sciences, Shanghai 200031, China

Pan Li and Lijun Zhang contributed equally to this work

To whom correspondence may be addressed. Tel: +1-86-10-6273-4163, Email: swu@cau.edu.cn

Correspondence may also be addressed to Xuguang Du. Tel: +1-86-10-6273-3075, Email: xuguangdu@cau.edu.cn

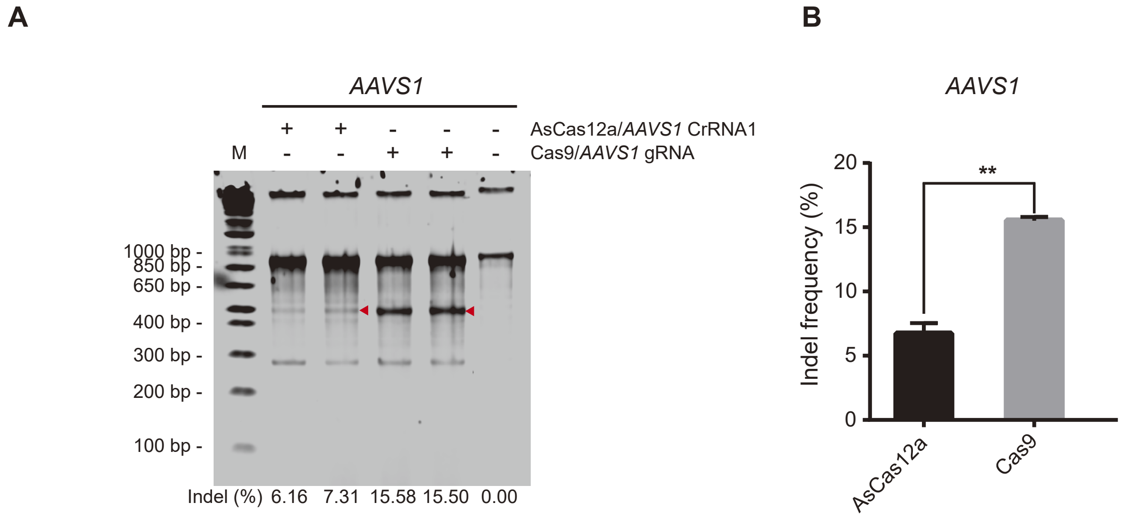


**Supplemental Figure S1.** The verification and comparison of the target efficiency between the Cas9 and Cas12a at the *AAVS1* locus. T7E1 analyses showing that the gRNA of Cas9 is more efficient than that of Cas12a when the same *AAVS1* site was targeted.The result was presented as mean ± SD, n=2, * $P < 0.05$, ** $P < 0.01$, *** $P < 0.001$, unpaired Student's t-test.

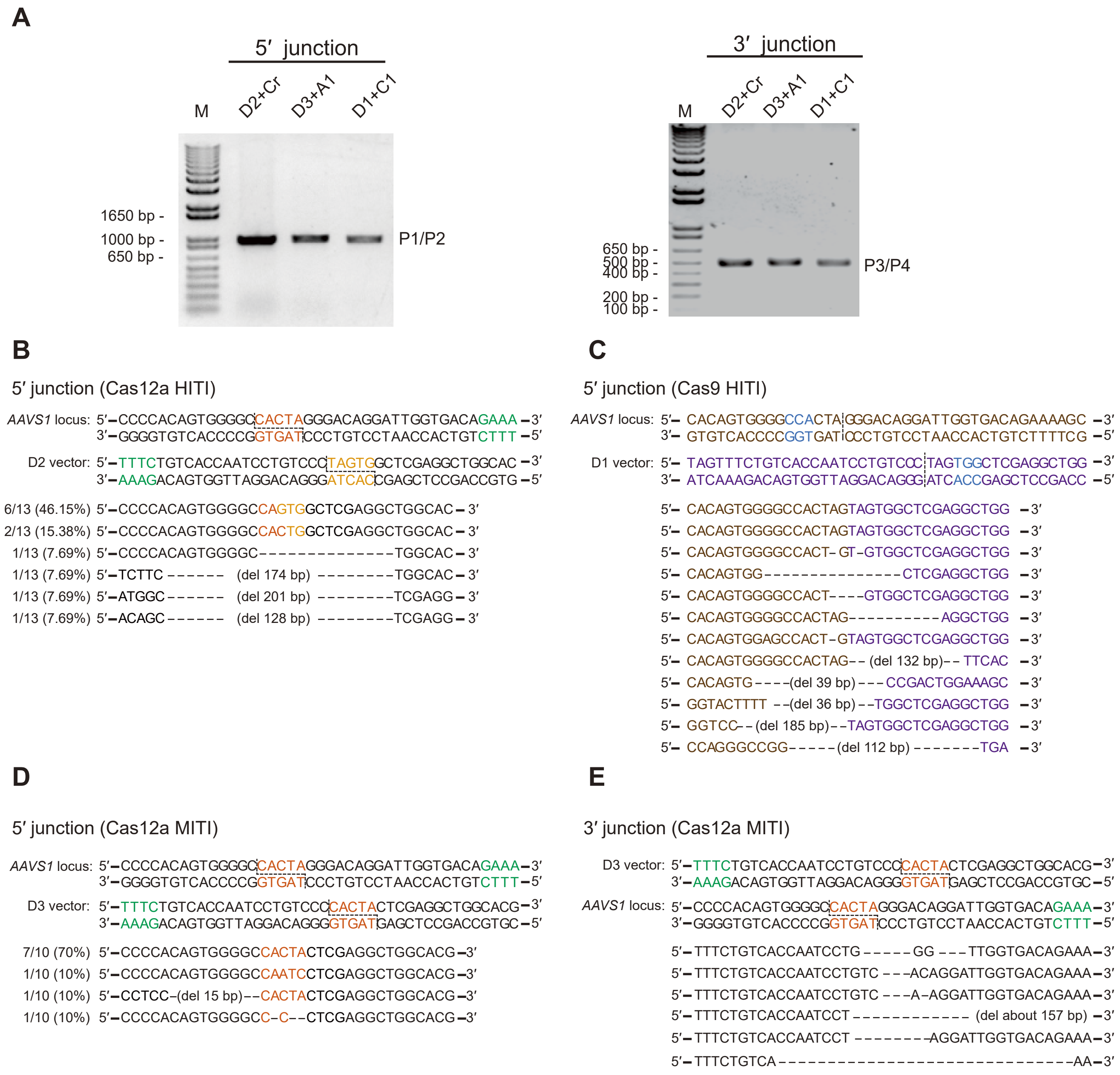


**Supplemental Figure S2.** Analyze the validity and accuracy of two junctions. **(A)** PCR analysis of 5' and 3' junctions of cells with Cas12a HITI, Cas12a MITI, and Cas9 HITI targeted integrations at the *AAVS1* locus in HeLa cells. Genomic PCR products amplified from pooled HeLa cells transfected with three groups of plasmids. The first group contained the Cas12a HITI donor (D2), *AAVS1* CrRNA1 and the Cas12a targeting vector (Cr); the second group included the Cas12a MITI donor (D3), *AAVS1* CrRNA1, *AAVS1* CrRNA1.1, and the Cas12a targeting vector (A1); and the third group had the Cas9 HITI donor (D1), *AAVS1* gRNA1 and Cas9 targeting vector (C1). M is the 1kb plus ladder maker. **(B)** The representative TA cloning sequence analysis at the 5' target junction of *AAVS1* targeted integration events mediated by Cas12a HITI strategy after PCR-based amplification. **(C)** The representative TA cloning sequence analysis at the 5' target junction of *AAVS1* targeted integration events mediated by Cas9 HITI strategy. **(D and E)** The representative TA cloning sequence analysis at the 5' and 3' target junction of *AAVS1* targeted integration events mediated by Cas12a MITI strategy.

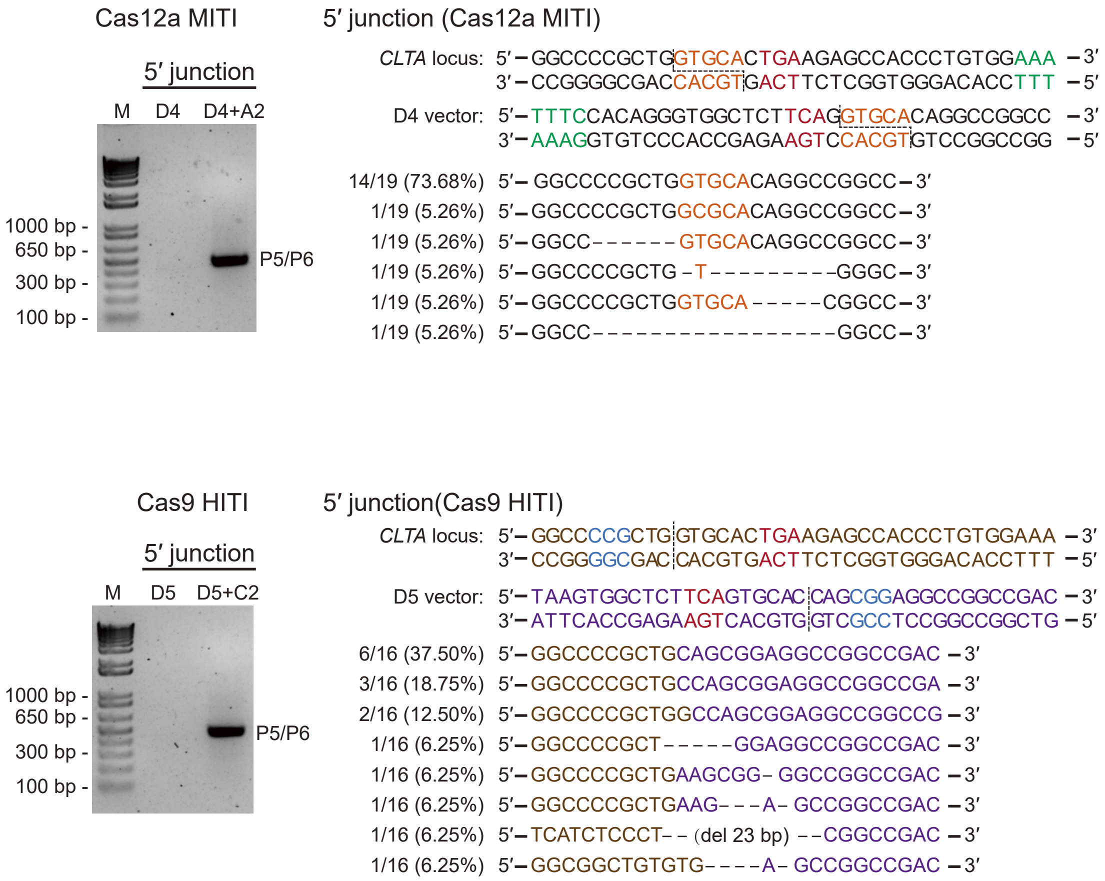


**Supplemental Figure S3.** Detection of target integration at the 5' junction at the *CLTA* locus. Genomic PCR products amplified from pooled HEK293T cells transfected with *CLTA* donor and the corresponding Cas12a or Cas9 targeting vector and the TA cloning sequence analysis at the 5' junction of *CLTA* targeted integration events after PCR-based amplification. A2 represents the Cas12a co-expression plasmid with the *CLTA* CrRNA array. C2 represents the *CLTA* gRNA and Cas9 co-expression plasmid. P5 and P6 primers are utilized to amplify the 5' junction. The right panel is the representative TA cloning sequence results of the 5' target junction of *CLTA* integration using the Cas12a MITI or Cas9 HITI strategy.

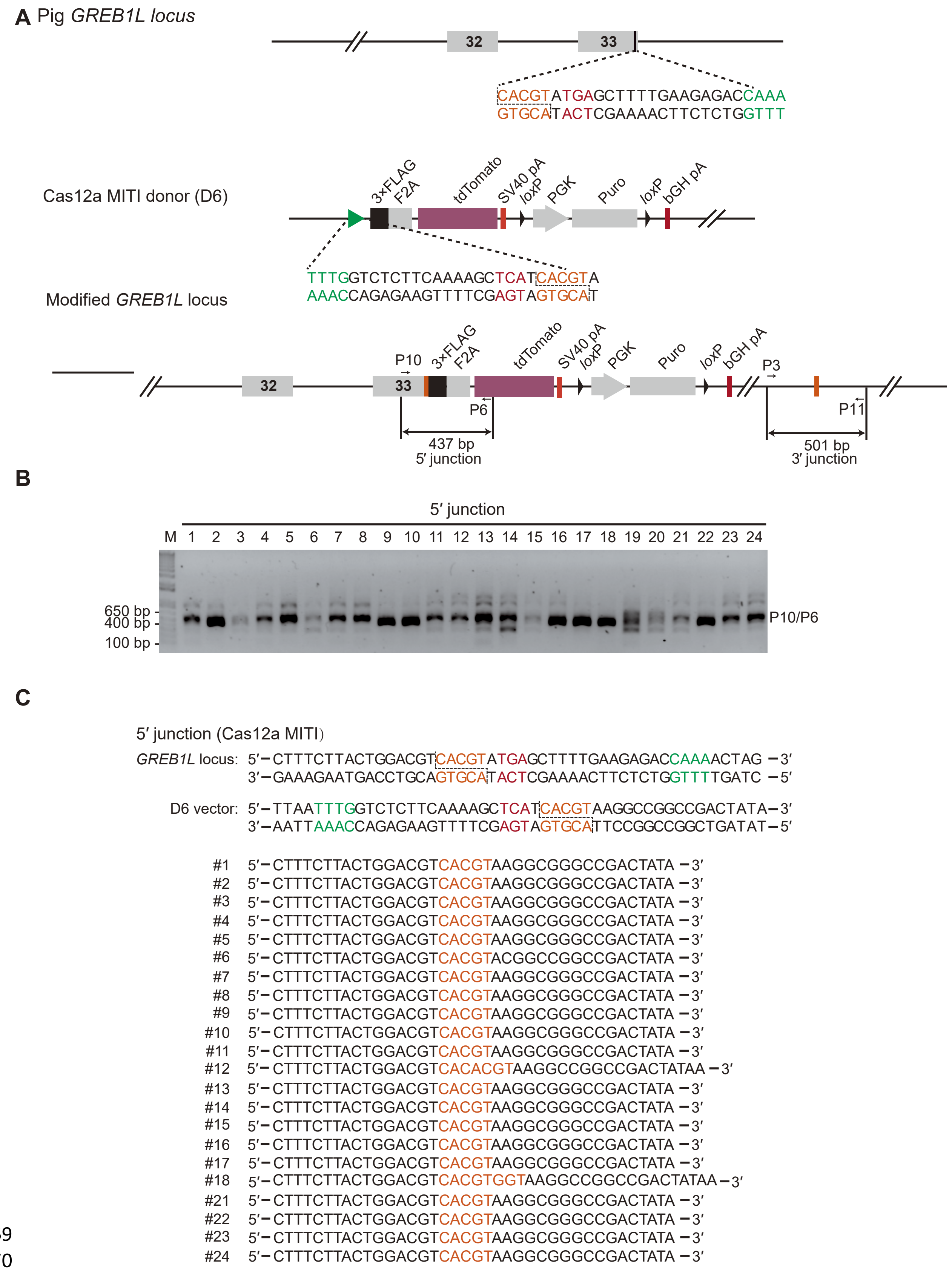


**Supplemental Figure S4.** Tagging the *GREB1L* gene in pig fetal fibroblasts (PFFs) cells using the MITI approach. **(A)** Strategy for targeting *GREB1L* locus in PFF cells. **(B)** PCR identification of positive PFF clones bearing predicted integration of 3×FLAG-F2A-tdTomato. **(C)** The 5' junction sequences of positive PFF clones.

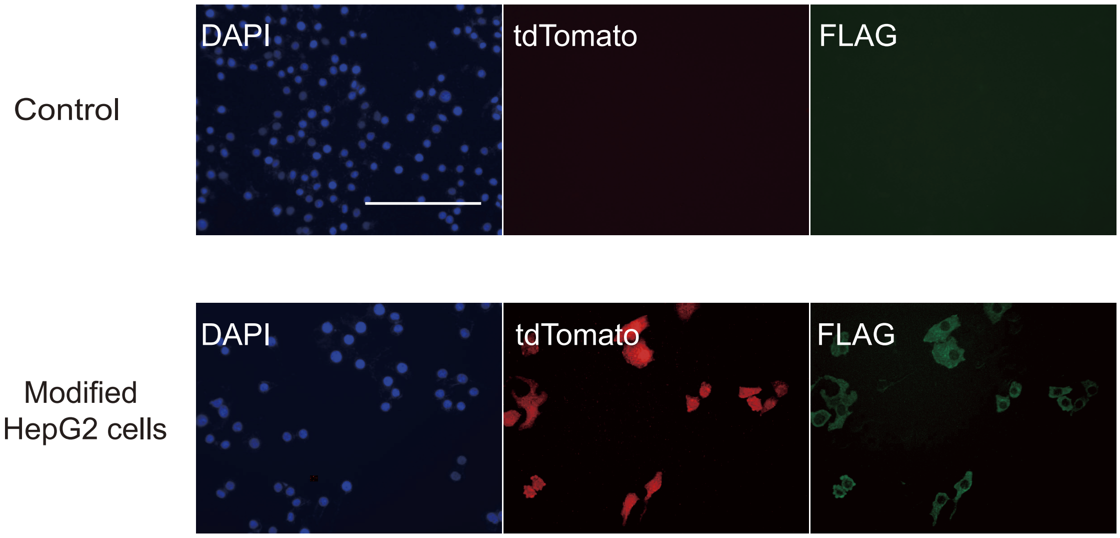


**Supplemental Figure S5.** Immunostaining results of tdTomato positive HepG2 cells. The tdTomato positive HepG2 cells bearing 3×FLAG-2A-tdTomato integration in *CLTA* were fixed, stained with anti-FLAG antibody and examined by fluorescence microscopy. Scale bar, 200 μm

**Supplementary Tables**

**Supplementary Table S1**. The primers for donor construction. All sequences are in the 5' to 3' direction

| Primer name | Sequence |
|---|---|
| D1/D3-linker-F | CTAGTTTCTGTCACCAATCCTGTCCCTAGTGGC |
| D1/D3-linker-R | TCGAGCCACTAGGGACAGGATTGGTGACAGAAA |
| D2-linker-F | CTAGTTTCTGTCACCAATCCTGTCCCCACTAC |
| D2-linker-R | TCGAGTAGTGGGGACAGGATTGGTGACAGAAA |
| D4-linker-F | TATGttaattaaTTTCCACAGGGTGGCTCTTCAGGTGCAcAGGCCGG |
| D4-linker-R | CCTgTGCACCTGAAGAGCCACCCTGTGGAAAttaattaaCA |
| D5-linker-F | TATGttaattaaGTGGCTCTTCAGTGCACCAGCGGAGGCCGG |
| D5-linker-R | CCTCCGCTGGTGCACTGAAGAGCCACttaattaaCA |
| D6-linker-F | TATGttaattaaTTTGGTCTCTTCAAAAGCTCATCACGTaAGGCCGG |
| D6-linker-R | CCTtACGTGATGAGCTTTTGAAGAGACCAAAttaattaaCA |
| D7-linker-F | CGCGCCAGGATCTCTGGCTCCATCGTAAGCAAAACGCGTGTCGACA |
| D7-linker-R | GATCTGTCGACACGCGTTTTGCTTACGATGGAGCCAGAGATCCTGG |

**Supplementary Table S2**. The primers for CrRNAs construction. All oligos are in the 5' to 3' direction.

| Primer name | Sequence |
|---|---|
| *AAVS1*-CrRNA1-F | agatTGTCACCAATCCTGTCCCTAGTG |
| *AAVS1*-CrRNA1-R | aaaaCACTAGGGACAGGATTGGTGACA |

| | |
|---|---|
| *AAVS1*-CrRNA1.1-F | agatTGTCACCAATCCTGTCCCCACTA |
| *AAVS1*-CrRNA1.1-R | aaaaTAGTGGGGACAGGATTGGTGACA |
| *AAVS1*-sgRNA1-F | CACCGTCACCAATCCTGTCCCTAG |
| *AAVS1*-sgRNA1-R | AAACCTAGGGACAGGATTGGTGAC |
| *CLTA*-CrRNA-array-F | agatCACAGGGTGGCTCTTCAGTGCACaatttctactcttgtagatCACA GGGTGGCTCTTCAGGTGCA |
| *CLTA*-CrRNA-array-R | aaaaTGCACCTGAAGAGCCACCCTGTGatctacaagagtagaaattGT GCACTGAAGAGCCACCCTGTG |
| *CLTA*-sgRNA-F | CACC GTGGCTCTTCAGTGCACCAG |
| *CLTA*-sgRNA-R | AAAC CTGGTGCACTGAAGAGCCAC |
| *GREB1L*-CrRNA-array-F | agatGTCTCTTCAAAAGCTCATACGTGaatttctactcttgtagatGTCT CTTCAAAAGCTCATCACGT |
| *GREB1L*-CrRNA-array-R | aaaaACGTGATGAGCTTTTGAAGAGACatctacaagagtagaaattCA CGTATGAGCTTTTGAAGAGAC |
| *AAVS1*-CrRNA-array-F1 | agatTGTCACCAATCCTGTCCCTAGTGAATTTCTACTCTTGT AGATTGTCACCAATCCTGTCC |
| *AAVS1*-CrRNA-array-F2 | CCACTAaatttctactcttgtagatCTTACGATGGAGCCAGAGAGGAT aatttctactcttgtagatCTTACGATGGAGCCAGAGATCCT |
| *AAVS1*-CrRNA-array-R1 | aaaaAGGATCTCTGGCTCCATCGTAAGatctacaagagtagaaattATC CTCTCTGGCTCCAT |

| | |
|---|---|
| | CGTAAGatctacaagagtagaaattTAGTGGGGACAGGATTGGTGAC |
| *AAVS1*-CrRNA-array-R2 | AATCTACAAGAGTAGAAATTCACTAGGGACAGGATTGGT GACA |

**Supplementary Table S3**. Primers used for Knock-in detection

| Primer name | Sequence |
|---|---|
| P1 | TGCCATCTCTCGTTTCTTAGGATG |
| P2 | cagaTcgataaaacacatgcgtcaattt |
| P3 | GCGTTTCGGTGATGACGGTG |
| P4 | CTGCCAAGCTCTCCCTCCCAG |
| P5 | GGGACAAATAGGCAGTTGCT |
| P6 | tcctcgcccttgctcaccat |
| P7 | CTCTGAATGCCAGGGAGAAC |
| P8 | TCTGTTCCACATACACTTCATTC |
| P9 | CCCGGTGCCTGAGATaaacG |
| P10 | CGGCTGTCACATCTTGGTTT |
| P11 | TCCAAAGCATCTCCTCAGGC |
| P12 | CAGGACGGGGCTGGCTACTG |

**Supplementary Table S4**. Primers used for T7E1 assay

| Primer name | Sequence |
|---|---|
| *AAVS1*-T7E1-sur-F | TGCCATCTCTCGTTTCTTAGGATG |

| | |
|---|---|
| *AAVS1*-T7E1-sur-R | CTGCCAAGCTCTCCCTCCCAG |
